# Simple Determination of p-Cresol in Plasma Samples Using Fluorescence Spectroscopy Technique

**DOI:** 10.22037/ijpr.2020.114330.14799

**Published:** 2021

**Authors:** Milad Moradi, Jafar Soleymani, Hamid Tayebi-Khosroshahi, Maryam Khoubnasabjafari, Abolghasem Jouyban

**Affiliations:** a *Pharmaceutical Analysis Research Center, Tabriz University of Medical Sciences, Tabriz, Iran. *; b *Liver and Gastrointestinal Diseases Research Center, Tabriz University of Medical Sciences, Tabriz, Iran. *; c *Immunology Research Center, Tabriz University of Medical Sciences, Tabriz, Iran. *; d *Kidney Research Center, Tabriz University of Medical Sciences, Tabriz, Iran. *; e *Biotechnology Research Center,* *Tabriz University of Medical Sciences, Tabriz, Iran. *; f *Tuberculosis and Lung Diseases Research Center, Tabriz University of Medical Sciences, Tabriz, Iran. *; g *Kimia Idea Pardaz Azarbayjan (KIPA) Science-Based Company, Tabriz University of Medical Sciences, Tabriz, Iran.*

**Keywords:** Chronic kidney disease, p-cresol, Routine analytical method, Fluorescence, Biomedical analysis

## Abstract

The development of simple, fast, cheap and reliable analytical methods for tracing biological indicators is demanded through clinical investigations. Herein, we developed, for the first time, a cheap and specific method for the extraction and quantification of p-cresol (pC) in real plasma samples of chronic kidney disease (CKD). Plasma samples were prepared by hydrolyzing in an acidic medium to convert pCS (p-cresol sulfate) and p-Cresol glucuronide (pCG) to pC. Next, proteins of plasma samples were precipitated and then pC was extracted by acetonitrile (ACN) and saturated NaCl (as salting-out agent). Finally, fluorescence emissions were measured at λ_ex_/λ_em _= 280/310 nm. The specificity of the method was checked by testing various possible interfering agents. The obtained results revealed a specific determination of pC. Under optimal conditions, a linear range was detected from 0.5 to 30 µg/mL of pC with a lower limit of detection (LLOQ) of 0.5 µg/mL. The reliability of the method was checked by calculating the repeatability, selectivity, and accuracy of the developed method for pC determination in plasma samples. The application of the developed method was investigated for the detection of pC in a number of CKD patients. Due to the simplicity and selectivity, the developed method could be applied for routine analysis of pC concentrations in the plasma samples of CKD patients. In addition, the developed method showed great potential for developing a point-of-care testing (POCT) device.

## Introduction

p-Cresol (4-methylphenol, CH_3_CH_4_OH, pC) is a product of bacterial metabolism of some amino acids (phenylalanine, tyrosine, and phenol) in the intestine. p-Cresol sulfate (pCS) is the major form of pC (about 95%) in the blood which is produced in the liver and colon. p-Cresol glucuronide (pCG) is another form of pC with 3–4% of which produced through glucuronidation of pC, while about 0.5–1% of the produced pC is available in free form (1, 2) which has been shown to be toxic in vitro. This study examined the form in which p-cresol circulates and quantified its removal by hemodialysis. HPLC analysis of plasma from hemodialysis patients contained a peak whose mobility corresponded to synthetic p-cresol sulfate (PCS. The half-life time of pC in the body is about 1.5 h; however, its metabolites *i.e.* pCS and pCG are stable for days. In healthy persons, pCS and pCG are continuously removed from the body while uremic toxins were not efficiently removed in patients and subsequently accumulated in the blood. The serum levels of pC (or pCG and pCS) in healthy normal persons are varied from 3.65 to 56.06 µM (0.34 to 5.3 µg/mL) (3). pC and its metabolites are related to various diseases such as chronic kidney disease (CKD) (4), cardiovascular disease (5) and autism disorder (6). Early-stage diagnosis of pCs in the bloodstream can result in control and reducing the harmful secondary effects. To do this, the first attempt is to develop sensitive approaches for determining pCs in the blood. Obviously, simple, fast, and selective approaches are welcomed by clinical investigations. 

Although, information related CKD in Iran is limited because of the lack of a central reporting registry, there are some studies that revealed that the overall prevalence of CKD is higher than the global average prevalence (7, 8). Bouya *et al. *(9) reported that the overall prevalence of CKD was 15.14% where the prevalence of CKD in female and male cases were 18.80% and 10.83%, respectively. 

A survey of the literature revealed that various analytical techniques such as high-performance liquid chromatography (HPLC), gas chromatography (GC), electrochemical and capillary electrophoresis were used to determine pC in various biological samples. However, each of them has its own limitations. Despite the significant advantages of chromatography techniques, their uses are limited to the research and development (R&D) laboratories and they are hardly utilized in routine clinical investigations. High costs of instrumentation, longer analysis time, and demanding spectra interpretation (especially in mass spectroscopy (MS)-based techniques) are the main limitations of the chromatographic techniques. In addition, at least one extraction step is usually required during or before analysis to clean up analytes from possible interfering agents available in biological fluids (10)i.e., high-performance liquid chromatography (HPLC. Moreover, a derivatization step is usually needed to enhance the response of the instrument to the analyte (11). 

Despite the great ability of electrochemical approaches, these methods were not largely utilized in reliable clinical detection of analytes. Therefore, it is highly requested to design a reliable method with sufﬁcient sensitivity to detect pC in biological fluids. Optical techniques, especially molecular absorbance and fluorescence spectroscopic methods, are advantageous analytical detection approaches in clinical labs which have been applied for many years. Generally, optical techniques have inexpensive and fast detection protocols which their sensitivity and specificity are comparable with more complicated techniques.

Detection of any analytes in plasma sample usually needs sample preparation steps to precipitate protein contents and then extract analyte from the matrix. Various types have been applied for the extraction of different analytes from biological fluids. Although new advanced materials improved the analytical performances of the extraction approaches, the repeatability of the extraction procedures usually is questionable. In addition, the repeatability of the synthesis of new advanced materials is not acceptable. Although liquid-liquid extraction (LLE) is an old method for extraction, it is one of our choices for routine analyte detection. LLE provides simple, low cost, relatively high enrichment factor, and good reproducibility. 

Salt-assisted liquid-liquid extraction (SALLE) is a type of LLE that has been largely applied for the extraction of pharmaceuticals in various biological fluids. In SALLE, a salt (such as NaCl, KCl, K_2_CO_3_, *etc*.) is added to the water-miscible organic solvent mixture to achieve a separation between aqueous and organic phases. For example, Wu *et al. *extracted a new drug candidate from a human plasma sample using ammonium acetate as a salting-out agent (12)only inorganic salts were evaluated and implemented as the salting-out reagents. A potential concern of the method for the subsequent LC–MS analysis of biological samples was that a portion of the added salt (typically of high concentration. Noche *et al.* determined five non-steroidal anti-inflammatory drugs (NSAIDs), including ibuprofen, clofibric acid, ketoprofen, naproxen, and diclofenac in the water samples by gas chromatography-mass spectroscopy (GC-MS) technique in which a SALLE approach was used to concentrate the drugs before injection to the GC-MS (13). Application of the SALLE approach in bio-analysis was comprehensively explained in a review paper reported by Tang *et al. *(14). Generally, SALLE possesses some advantages over the other extraction approaches (LLE and SPE) including high clean-up factor, simulations extraction of multiple analytes and high enrichment factor (15). 

This work aimed to develop a simple and reliable florescence-based method for the detection of pC in plasma samples. The developed approach was utilized to determine pC contents of plasma samples of CKD patients. pC contents were extracted *via* a simple extraction step *i.e.* SALLE, without any derivatization step. The developed method is the first fluorescence method for the quantification of pC in plasma samples. The analytical performances were validated in plasma samples, where results revealed that this method possesses relatively high sensitivity, selectivity, and reproducibility in the plasma environment. 

## Experimental

*Materials and Instruments*

p-Cresol was obtained from Sigma (St. Louis, MO). Hydrochloride acid (HCl) and sodium hydroxide (NaOH) were purchased from Scharlau (Barcelona, Spain). Acetonitrile (ACN) and NaCl were provided from Merck (Darmstadt, Germany). Deionized water was used to prepare all solutions. All substances were used as received without any purification steps. Healthy plasma samples were provided from Iranian blood transfusion organization (Tabriz, Iran) and patient samples were collected from Imam Reza hospital (Tabriz, Iran) under the approval code of IR.TBZMED.REC.1397.442. 

Fluorescence spectra were recorded on a JASCO FP 750 spectrofluorometer (Tokyo, Japan) equipped with a 150 W xenon lamp, using 1.0 cm quartz cells. The emission and excitation slits were adjusted to 5 nm. Effect of temperature was checked using a Peltier thermostated cell holder (JASCO, Japan). The excitation and emission wavelengths of pC were adjusted at 280 nm and 310 nm, respectively. pH determinations were measured using a Metrohm744 pH meter (Herisau, Switzerland). 

*Preparations of standard stock solution and plasma samples*

A stock solution (1000 μg/mL) of pC was prepared for plotting calibration curve and quality control (QC) assays by dissolving the proper quantity of pC in ACN and then stored at room temperature. It is only necessary to dilute the stock solution to prepare the next working solutions. Plasma samples of healthy people were spiked with various concentrations of pC to plot the calibration curve. Firstly, healthy plasma samples are thawed and then vortexed to homogenize the constituents. Next, to the 0.1 mL of plasma, appropriate concentrations of pC are added and shaken. Finally, the provided spiked plasma samples are utilized for the construction of calibration curves and other validation purposes. 

*Sample preparation and extraction procedure*

A liquid-liquid extraction approach was used to extract pC from plasma samples. 100 µL of plasma samples were added to a 2-mL microtube and then 25 µL HCl (6M) was added and heated for 2 min at 80 °C for acid-catalyzed hydrolysis of pCS and PCG. Next, samples have cooled to room temperature and then 900 µL of ACN was added and shaken for about 15 min. Then, 0.5 mL of saturated NaCl was added and shaken for another 15 min. Finally, the mixture was centrifuged for 10 min at 10000 rpm and fluorescence emissions of A organic phase (1 mL) were measured at λ_ex_/λ_em _= 280/310 nm. 

*Method validation*

Method validation is a way used to approve that a developed analytical method used for the detection of a specific method is appropriate for its planned use. Method validation results could be utilized to evaluate the reliability of an analytical for the detection of a specific analyte. In this work, the analytical performance and stability of the method were implemented in accordance with food and drug administration (FDA) guidelines in terms of linearity, lower limit of quantification (LLOQ), the limit of detection (LOD), the limit of quantification (LOQ), correlation coefficient, precision, specificity, accuracy, recovery, and stabilities (16).

The specificity of the method was verified by comparing interferences of possible coexisting agents to the emission intensity of the pC at 310 nm *via* spiking of interfering agents to the drug-free plasma samples. As the FDA rule, interference of the agents should be lower than 20%. The precision and accuracy are important to control whether the developed method is appropriate for testing an analyte in real samples. The interday and intraday precision and accuracy were implemented in spiked plasma at three QC levels of low (LQC), medium (MQC), and high (HQC). The recovery of the method was tested by measuring the fluorescence emission of pC at LQC, MQC, and HQC plasma samples and then calculating the concertation of pC in plasma samples by the calibration curve. 

The stability of the developed was tested at LQC and HQC concentrations. Bench-top stability assay was evaluated by storing plasma samples at room temperature for at least 12 h. Freeze-thaw stability assay was confirmed by repeating freeze and thaw of plasma samples for three cycles, from -20 °C to room temperature. The stock solution stability of pC at 4 °C and room temperature for 24 h was also evaluated.

## Results and Discussion

*Spectroscopic study*

**Figure 1:**
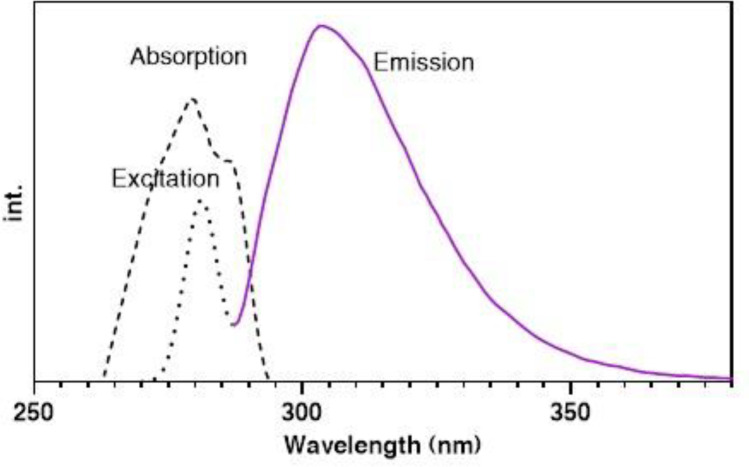
Absorption (dashed line), excitation (dotted line) and emission (solid line) spectra of pC (5 µg/mL) in absolute ACN (room temperature).

The absorption spectrum of pC was determined using an ultra-violent technique, where results showed that the absorption peak is around 280 nm (Figure 1). To approve the absorption (excitation) and emission wavelengths, the excitation and fluorescence emissions were investigated by the fluorimetry technique. As Figure 1 shown, pC excites at 280 nm and emits fluorescence at 310 nm. The fluorescence emission is linear with pC concentrations. The same excitation and emission were observed in both plasma and standard media, approving the independence of the fluorescence emission in complex plasma matrices. 

*Factors affecting the fluorescence intensity of the system*

**Figure 2:**
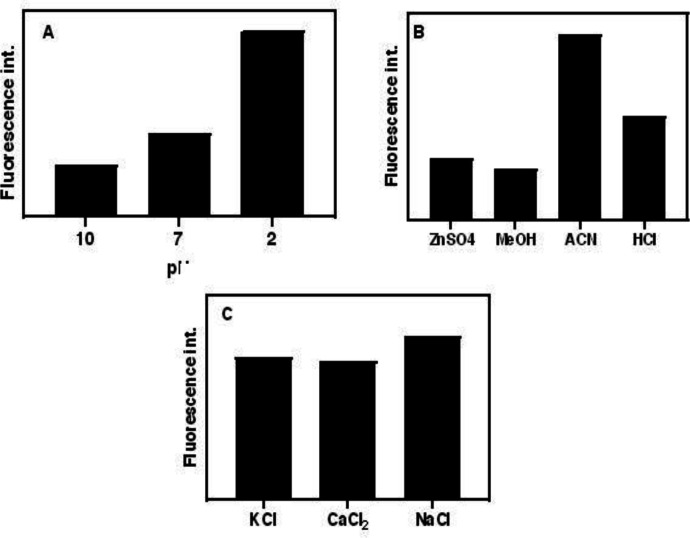
(A) Effect of pH on the hydrolysis of pCS and PCG to pC, (B) effect of type of protein precipitant and (C) salting-out agent fluorescence emission. (conditions: (A) HCl, NaOH, and PBS concentrations are 0.01 M, and temperature, 80  C (B) ZnSO_4_ in saturated form, ACN, MeOH in absolute form and HCl 36%, room temperature, and (C) all salts are saturated at room temperature)

To regulate the best conditions for increasing the sensitivity of the method toward a specific analyte, the optimization step of the parameters is of great importance. Firstly, the proper slit sizes of emission and excitation were selected. It is important to select the proper slit because of its effect on the fluorescence of analyte and coexisting agents. In this study, 5 nm was selected for both excitation and emission slits. Due to the fact that pH has the main effect on the hydrolysis of pCS to pC, the acidity and basicity value of the hydrolysis reaction were also investigated. Accordingly, the same concentrations of pC at three pH values (acidic, neutral and basic) and their blanks were prepared and then fluorescence emissions were measured. As Figure 2 shows, the acidic medium showed the highest fluorescence intensity, where the conversion of pCS to pC was strongly dependent on the pH of medium and reached a maximum value at a pH value of about 1.5. The pHs of solutions were adjusted by HCl, NaOH and phosphate buffer solution (PBS). 

Various agents were checked for the effective protein precipitation of plasma samples. ACN was selected as the best protein precipitant and then the ACN/plasma ratio was optimized as 1:9. Although the higher sample volume means the higher extraction of the analyte, fluorescence emissions of coexisting agents overlap with pC fluorescence spectrum. The effect of reaction time was also tested. Obtained results revealed that 2 min is the optimal reaction time to convert all pCSs to pC. In lower and higher time periods, the fluorescence intensities dropped because of reaction incompletion and possible secondary reactions of pC, respectively. Also, the effect of shaking time was optimized and 15 min selected for completion of extraction of pC from plasma samples.

The salting-out ability of different salts was tested by introducing various salts into the ACN-extracted solution. Obtained results proposed NaCl as the most efficient agent for convincing salting-out force to the pC molecules for migration from the aqueous phase to the organic phase. Further experiments showed that saturated NaCl had the maximum effect on the extraction of pC.

After optimizing the parameters, the effect of time of analysis was investigated on the fluorescence emission of pC. The results obviously showed that fluorescence emission was constant for the first 5 min. All measurements were carried out immediately after the extraction step. Room temperature was selected as the optimal temperature for recording the fluorescence spectra since temperature control systems are not available in all fluorescence sets.

*Analytical figures of merit*

**Figure 3:**
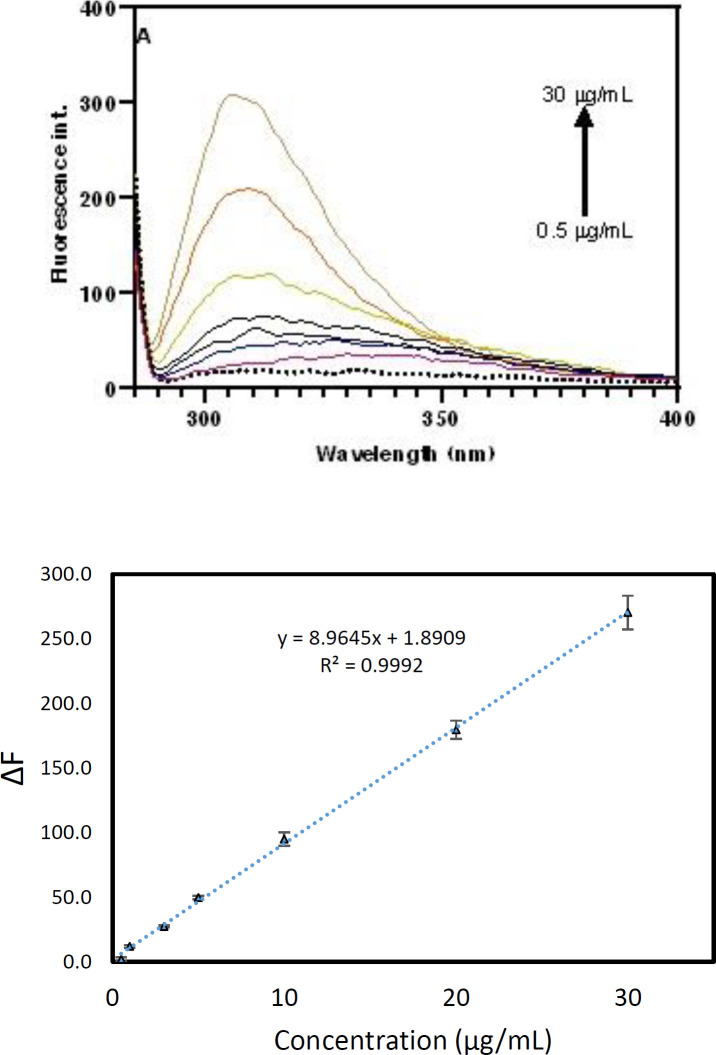
(A) Fluorescence spectra and (B) corresponded calibration curve of pC at various concentrations from 0.5 to 30 µg/mL.

A simple fluorescence method was developed for the measurement of pC in plasma samples based on the intrinsic fluorescence emission of pC at 310 nm. After optimization of the influence of parameters, the calibration curve was plotted in the plasma matrix to discover the dynamic range and other figures of merit. To plot the calibration curve, various concentrations of pC were prepared and then linear range, LLOQ, precision and accuracy of the method were calculated. In the plasma matrix, various concentrations of pC were added to the 100 µL sample and then extracted as reported in subsection 2.3. The ﬂuorescence emission was enhanced by increasing pCs ranging from 0.5 to 30 µg/mL. The fluorescence difference between blank and analyte was regarded as a signal to plot the calibration curve. The regression equation for plasma was ∆F = 8.9645 (C (µg/mL)) + 1.89 with R^2^ of 0.9992 and the relative standard deviation (RSD%) of 1.2%. LOQ, LLOQ and LOD of the developed approach were 0.6, 0.5 µg/mL and 0.2 µg/mL, respectively. The high Y-intercept of the developed method is mainly caused by the fact that serum samples of healthy subjects possess some pC (or pCG and pCS) in the range from 3.65 to 56.06 µM (0.34 to 5.3 µg/mL) (3). Figure 3 illustrated the calibration curve and corresponded fluorescence spectra.

A summary of figures of merit of the developed method and comparison with the previously reported methods for the determination of pC are collected in Table 1, respectively (10, 17-26). To date, the fluorescence technique has not been utilized for the quantification of pC in biological samples. Compared with the previously reported approaches, the developed method has a relatively simple procedure with comparable sensitivity. However, better sensitivities were obtained with more expensive and complex techniques such as liquid chromatography-mass spectroscopy/mass spectroscopy (LC-MS/MS) and GC-MS/MS. Although reported methods can provide better sensitivities, they often need complicated sample preparation steps with consuming hazardous solvents and high-cost instruments. Typically, highly skilled staff are required to work with GC-MS/MS and LC-MS/MS and interpret the results. 

*Speciﬁcity, repeatability and accuracy of the method*

The specificity of a method is a crucial topic in the detection of an analyte in biological media. Specificity is defined as the response of a developed method towards a specific analyte in the presence of possible coexisting agents. In other words, it refers to the unequivocal determination of a proper amount of an analyte in the presence of various coexisting agents. Specific methods detect and differentiate the target analyte from any other non-target agents in the presence of various interfering macro-molecules and ions.

To quantitatively test the specificity of the developed method for the determination of pC, pC was determined in the presence of several agents. However, in the case of the analysis of the patient’s fluids, the effect of consumed pharmaceuticals on the signal of pC should be checked. Table 2 lists the effects of prescribed drugs on the fluorescence intensity of the probe. Results showed that the developed method was highly specific towards pC in plasma samples. Some interfering agents have changed the original fluorescence intensity of pC, which mainly corresponded to the higher concentrations of the agents, approving the specificity of the developed method. 

According to the FDA guidelines, repeatability of a method is the ability of the developed method to produce similar results for a given concentration of analyte, which is mainly associated to the precision of the method and stated in RSD%. The repeatability of the developed method was investigated by three concentrations of pC from three quality control (QC) levels of low (LQC), medium (MQC) and high (HQC). After processing extraction steps, fluorescence emissions were recorded on the same day (or different days) and the repeatability was expressed in RSD%. Repeatability is expressed in two forms of interday and intraday. Results revealed that the developed method behavior is repeatable for both intraday and interday assays and all RSD% values were reported lower than 15% as allowed by FDA. The repeatability of the developed method is reported in Table 3.

The accuracy of a method for a specific analyte is the maximum allowed differences between the actual and the calculated values. Based on, the fluorescence emission of the three concentrations of pC from three points of the calibration curve was measured and then the recovered amounts of pC were calculated by calibration curve to estimate the accuracy of the method for pC determination in plasma samples. The recoveries of the developed method for 1, 5 and 20 µg/mL concentrations of pC were 112.8, 107.0 and 99.2%, respectively, approving the acceptable accuracy of the developed method for the determination of pC in plasma samples (Table 4). 

*Stability*

The stability of an analyte in a biological matrix is an important factor that affects the obtained results. The room temperature and freeze-thaw stability are two important stability factors and determined as follows. To check room temperature stability, three concentrations of pC were prepared and left for 24 h at room temperature and then fluorescence intensities were measured with the developed approach (Table 5). For freeze-thaw stability, three concentrations of pC were prepared and then freeze-thawed for three cycles. After the third cycle, samples were immediately analyzed with the developed method (Table 5). Results showed that both stabilities are acceptable and their standard deviations are lower than 20% as allowed by FDA guidelines (15).

Also, stock solution stability of pC was measured at room temperature and in a refrigerator. To do this, 5 µg/mL of pC was prepared in two sets and one set was left at room temperature and another stored in a refrigerator for 24 h. Then, the stability of pC was tested and compared with a freshly prepared solution of pC. Results approved that pC stock solution is stable at both conditions (Table 6).

*Application of the developed method for pC detection in patients’ real samples analysis*

The application of the developed method for the determination of pC concentration in patients’ plasma samples was evaluated under optimal conditions. Prior to analysis with the method, blood samples were taken from CKD patients from Imam Reza Hospital and then centrifuged to separate red blood cells. Then, they were stored at -20 °C till analysis with the developed method. Under optimal conditions, samples were thawed and fluorescence emissions were recorded and finally, concentrations were calculated using calibration curve equation. Obtained results are provided in Table 7. 

*Capability to be as a routine analytical procedure and a POCT device*

Daily routine analysis of biological agents is important to understand the patients’ condition and prescribe medicines with proper dosages. Reliable analytical methods with high specificity, precision, and accuracy can be routine analysis methods in clinical laboratories. To qualify an analytical method as a “routine analytical method”, it is important to check its performance in large numbers of biological samples. 

It is emphasized that the developed method could be applied as a “routine analytical method” for the determination of pC in plasma samples because of its favorable analytical features. The reported method is very simple, specific, sensitive, cheap and fast, which are the perquisites for a “routine analysis method” and also it showed high repeatability (interday and intraday) and stability. 

**Table 1:** Figures of merit of various strategies for pCs determination in biological samples

**Method**	**Sample**	**Approach**	**LOD** ^*^ ** (µg/mL)**	**Linear range (µg/mL)**	**Reference**
LC-FL	Serum	Extraction	0.14	0–50	(17)
GC-FID	Whole blood	Extraction	0.6	2–20	(18)
LC-FL	Plasma	Extraction	0.324	0.5–8.0	(10)
LC-FL	Urine	Extraction derivatization	0.018	0.01–540	(19)
UHPLC-MS/MS	Serum	Extraction	0.25	0.25–80	(20)
GC-MS	Serum	Extraction	LOD: 0.15 LOQ: 0.30	-	(21)
LC-MS/MS	Plasma	Extraction	0.85 ng/mL	0.85–200	(22)
HPLC	Serum	Extraction	0.25	0.5–50.0	(23)
UPLC-MS/MS	Serum	Extraction	0.05	0.05–5	(24)
UPLC-FL	Serum	Extraction	0.005	0–4.52	(25)
Electrochemical	Serum	-	1.1	108–2700	(26)
FL	Plasma	Extraction	0.5 (LLOQ) 0.2 (LOD) 0.6 (LOQ)	0.5–30	This work

^*^LOD: limit of detection; LC-FL: liquid-chromatography-fluorescence; GC-FID: gas chromatography-flame ionization detector; UHPLC-MS/MS: ultra-high performance liquid-chromatography-mass spectrometry-mass spectrometry; GC-MS: gas chromatography-mass spectrometry; LC-MS/MS: liquid-chromatography-mass spectrometry-mass spectrometry; UPLC-FL: ultra-performance liquid-chromatography-fluorescence; FL: fluorescence.

**Table. 2:** Effects of common prescribed pharmaceutical drugs in CKD patients on the ﬂuorescence emission intensity of pC

**Drug**	**Amounts of co-prescribed drug and tested concentrations (µg/mL)**	Intensity*	**Signal Change (%)**
Prozosin	0.05	56	102.3
Amlodipine	0.02	57	104.2
Simvastatin	0.03	57	103.4
Folic acid	0.015	49	89.3
Propranolol	0.3	60	109.9
Metoprolol	0.5	63	113.7
Furosemide	6.0	59	106.4
Bisoprolol	0.1	58	106.1
Captopril	0.5	58	104.6
Valsartan	6.0	56	102.3
Nitroglycerin	0.015	51	92.0
Lisinopril	0.14	51	92.7
Gemfibrozil	25.0	54	98.5
Enalapril	0.1	54	98.5
Vazonidin	0.02	56	101.1
Rouuvatatin	0.16	52	93.9
Lovastatin	1.0	56	102.3

**Table 3:** Precision and accuracy of the determination of pC for at least three replicates

**Nominal concentration (µg/mL)**	**Intraday precision (RSD%)**	**Interday precision (RSD%)**	**Intraday accuracy (RE%)**	**Interday accuracy (RE%)**
1.00	2.50	3.79	-12.83	-16.56
5.00	6.75	5.76	-7.02	-8.13
20.00	6.28	4.15	0.79	-5.34

**Table 4:** Recovery of the developed method for determination of pC in plasma samples

**Nominal concentration (µg/mL)**	**Found concentration (µg/mL)**	**Recovery (%)**	**Nominal concentration (µg/mL)**
1.0	1.13	112.8	1.00
5.0	5.35	107.0	5.00
20.0	19.84	99.2	20.00

**Table 5:** Details of the stability of the proposed method in plasma samples

**Nominal concentration** **(µg/mL)**	**Room temperature**		**Freeze-thaw**	
	**Mean found (µg/mL)**	**Accuracy** **(RE%)**	**Mean found (µg/mL)**	**Accuracy** **(RE%)**
1.0	1.1	-9.1	1.1	-9.1
5.0	5.5	-10.4	5.5	-9.6
20.0	20.2	-0.8	20.0	0.1

**Table 6:** Stock solution stability of pC

**Concentration (µg/mL)**	**Immediately analyzed (µg/mL)**	**24 h (RT** ^*^ **) (µg/mL)**	**24 h (4-8 C) (µg/mL)**
5	4.98	4.56	4.71

^*^room temperature.

**Table 7:** Obtained results for the determination of pC concentration in plasma real samples using the developed method

**Patient (#)**	**Gender**	**Age**	**Measured concentration (µg/mL)**
1	Female	84	1.71
2	Female	57	3.43
3	Female	50	3.21
4	Male	78	2.08
5	Male	65	3.10

## Conclusion

Despite the importance of pC, only a few analytical approaches were reported for its determination in biological samples. To the best of my knowledge, there is no report for the detection of pC in biological samples by fluorescence method. As Table 1 shows, the reported methods were mainly based on mass spectrometry, which is a high cost and time-consuming technique. 

For the first time, a simple spectrofluorometric approach was reported for the sensitive determination of pC in plasma samples without any derivatization steps. In this work, a simple liquid-liquid extraction was used to extract pC from plasma samples. The proposed approach showed high accuracy and repeatability with acceptable recoveries. The dynamic range and LLOQ of the developed method were 0.5 to 30 µg/mL and 0.5 µg/mL, respectively. The outstanding features of the method include simplicity, wide dynamic range, relatively high sensitivity, and excellent repeatability. The reported features propose the developed method as a reliable approach for tracing pC in CKD patient’s plasma samples. The results in real samples approved the reliability of the method for the routine determination of pC in clinical samples.
